# Oral Cancer Pain Includes Thermal Allodynia That May Be Attenuated by Chronic Alcohol Consumption

**DOI:** 10.3390/ph16040518

**Published:** 2023-03-31

**Authors:** Cara B. Gonzales, Jorge J. De La Chapa, Amol M. Patwardhan, Kenneth M. Hargreaves

**Affiliations:** 1 Department of Comprehensive Dentistry, UT Health San Antonio, School of Dentistry, San Antonio, TX 78229, USA; 2 Mays Cancer Center, UT Health San Antonio, San Antonio, TX 78229, USA; 3 Department of Anesthesiology and Pain Management, UT Southwestern Medical Center, Dallas, TX 75390, USA; 4 Department of Endodontics, UT Health San Antonio, School of Dentistry, San Antonio, TX 78229, USA

**Keywords:** oral squamous cell carcinoma, cancer-related pain, alcohol drinking habits, patient reported outcomes, analog pain scale, thermal pain

## Abstract

Background: Oral cancer is one of the most painful cancer types, and is often refractory to existing analgesics. Oral cancer patients frequently develop a tolerance to opioids, the mainstay of current cancer pain therapy, leaving them with limited therapeutic options. Thus, there is a great need to identify molecular mechanisms driving oral cancer pain in an effort to develop new analgesics. Previous reports demonstrate that oral cancer patients experience intense mechanical pain and pain in function. To date, no studies have examined thermal pain in oral cancer patients or the role that alcohol consumption plays in oral cancer pain. This study aims to evaluate patient-reported pain levels and thermal allodynia, potential molecular mechanisms mediating thermal allodynia, and the effects of alcohol consumption on patient-perceived pain. Methods: This study evaluated human oral squamous cell carcinoma (OSCC) cell lines for their ability to activate thermosensitive channels in vitro and validated these findings in a rat model of orofacial pain. Patient-reported pain in a south Texas OSCC cohort (n = 27) was examined using a visual analog scale (VAS). Covariant analysis examined variables such as tobacco and alcohol consumption, ethnicity, gender, and cancer stage. Results: We determined that OSCC secretes factors that stimulate both the Transient Receptor Potential Ankyrin type 1 channel (TRPA1; noxious cold sensor) and the Transient Receptor Potential Vanilloid type 1 channel (TRPV1; noxious heat sensor) in vitro and that OSCC-secreted factors sensitize TRPV1 nociceptors in vivo. These findings were validated in this cohort, in which allodynia to cold and heat were reported. Notably, subjects that reported regular alcohol consumption also reported lower pain scores for every type of pain tested, with significantly reduced cold-induced pain, aching pain, and burning pain. Conclusion: Oral cancer patients experience multiple types of cancer pain, including thermal allodynia. Alcohol consumption correlates with reduced OSCC pain and reduced thermal allodynia, which may be mediated by TRPA1 and TRPV1. Hence, reduced pain in these patients may contribute to a delay in seeking care, and thus a delay in early detection and treatment.

## 1. Introduction

Head and neck cancer pain is one of the most intense types of cancer pain, preceded only by ovarian and mesothelioma cancer pain [1,2]. Studies of patients with oral squamous cell carcinoma (OSCC) confirm that pain is frequently the primary presenting symptom, and may be a marker of progression of a premalignant lesion to a malignant lesion [3,4]. In addition, OSCC pain is not always proportionate to the tumor size. Relatively small tumors can elicit a disproportionate amount of pain, indicating that mere tumor mass and local tissue destruction are not the sole cause of OSCC pain [5]. Indeed, a number of studies demonstrate that tumors secrete factors that activate nociceptors found in the head and neck. These include endothelin-1 (ET-1), tumor necrosis factor alpha (TNFα), protease-activated receptor 2 (PAR2), and bone derived nerve factor [5,6,7,8,9,10,11]. Recently, Dubeykovskaya et al. (2022), determined that OSCC patients with nodal disease overexpress 40 genes with oncogenic and neuronal functions, many of which are reported in exosomes and provide credence to the concept that tumor secreted factors mediate oral cancer pain and are indicative of malignant transformation and metastatic potential [12].

OSCC patients with tongue and/or floor-of-mouth tumors report more pain than patients with tumors in other locations, such as the gingivae. Notably, OSCC pain is not only due to tumor location; rather it is also a result of the type of tumor located in the oral cavity [6]. For example, Chodroff et al. (2016) demonstrated that tongue cancer pain is also dependent upon cancer type [6]. An evaluation of feeding behavior, body weight, and mechanical allodynia using the colon cancer cell line HT29, the OSCC cell line HSC2, and oral keratinocyte cells (OKF6-Tert2) xenografted into the tongue of athymic nude mice showed that the OSCC cell line significantly reduced food intake, reduced body weight, and increased mechanical allodynia, whereas the colon cancer and oral keratinocyte cell lines had no effect on these parameters [6].

Lastly, studies demonstrate that oral cancer patients experience different types of cancer pain, but spontaneous pain and mechanical allodynia are the most prevalent [13,14]. The quality of the pain tested included sharp and aching pain sensations that occur both spontaneously and when in function, e.g., eating and speaking [13,14]. To date, no studies have been performed to determine if pain in response to thermal changes exists in oral cancer patients. Therefore, we performed in vitro assays analyzing OSCC-secreted factors for their ability to stimulate two thermosensitive ion channels, TRPA1 (activated by noxious cold) and TRPV1(activated by noxious heat), in cultured trigeminal ganglions (TG). Calcitonin Gene-Related Peptide (CGRP) is a neurotransmitter released from primary afferent neurons (C and Aδ) following painful peripheral stimuli [15,16,17]. To assess the activation of TG cultures, we measured the release of CGRP in response to OSCC conditioned media (CM) and unconditioned media (UCM). To evaluate the possible sensitization of these channels by OSCC-secreted factors, we pretreated with CM or UCM followed by known agonists (capsaicin (CAP) for TRPV1 and mustard oil (MO) for TRPA1). We then performed analyses of TRPV1 and TRPA1 sensitization in vivo using a rat model of orofacial pain. Lastly, we conducted a clinical pain study in OSCC patients using a visual analog scale based upon the University of California San Francisco (UCSF) Oral Cancer Pain Questionnaire [14], but with the addition of questions related to pain upon cold and heat exposure, and burning pain. Covariant analyses including stage, sex, alcohol, and tobacco usage were also assessed.

## 2. Results

### 2.1. OSCC-Secreted Factors Activate TRPA1 and TRPV1 Channels

**Figure 1:**
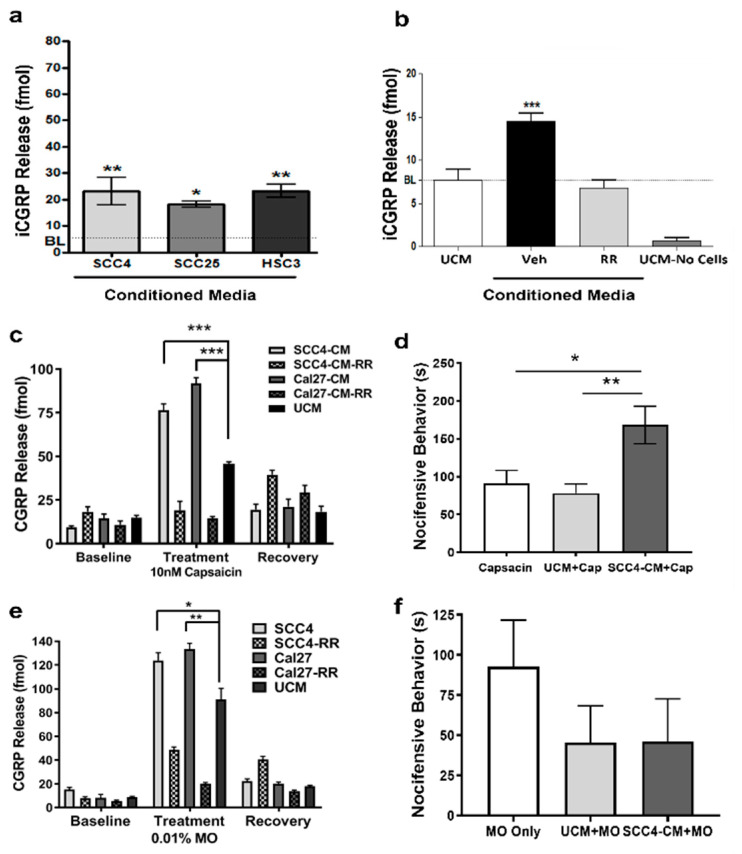
(**a**) Radioimmunoassay of CGRP release from primary cultured TGs in response to OSCC-derived CM compared to UCM. (**b**) SCC-4 CM stimulated CGRP release reversed by RR. (**c**) Radioimmunoassay of CGRP release from primary cultured TGs pretreated with OSCC-derived CM followed by 10 nM CAP. Baseline measurements are from OSCC-derived CM alone. Treatment measurements are from co-treatment of OSCC-derived CM with10 nM CAP, +/−RR. Recovery measurements following washes demonstrate vital TGs following treatment. (**d**) Eye-wipe studies measuring nocifensive behaviors in rats (n = 6) when exposed to OSCC-derived CM followed by 0.01% CAP compared to CAP alone and UCM plus CAP. (**e**) Radioimmunoassay of CGRP release from primary cultured TGs pretreated with OSCC-derived CM followed by 0.01% MO. Baseline measurements are from OSCC-derived CM alone. Treatment measurements are from the co-treatment of OSCC-derived CM with 0.01% MO, +/−RR. Recovery measurements following washes demonstrate vital TGs following treatment. (**f**) Eye-wipe studies measuring nocifensive behaviors in rats (n = 6) when exposed to OSCC-derived CM followed by 0.01% MO. * *p* < 0.05, ** *p* < 0.01, and *** *p* < 0.001.

We first tested the hypothesis that OSCC secrete factors that activate thermosensitive channels (e.g., TRPA1 and TRPV1) by measuring the release of CGRP from TG cultures in response to OSCC-derived CM. We determined that OSCC CM significantly activates TGs in vitro compared to control UCM (Figure 1a). When pretreated with the nonselective cation channel antagonist ruthenium red (RR), CGRP release returned to baseline (Figure 1b; *p* < 0.001).

To determine if tumor-secreted factors sensitize TRPV1 channels, TG cultures were pretreated with CM and UCM, followed by 10 nM CAP alone or 10 nM CAP in combination with RR (10 µM). Baseline measurements of CGRP release in response to CM and UCM were taken prior to treatments. Following treatments with CAP, cultures were washed and recovery measurements of CGRP release were taken to confirm the viability of TGs following treatments. Pretreatment with OSCC-derived CM followed by CAP induced a significant increase in CGRP release (compared to UCM) that was inhibited by RR (Figure 1c; *p* < 0.001). Recovered TGs continued to secrete baseline levels of CGRP following treatment with CAP. To validate that OSCC-secreted factors sensitize TRPV1 channels in vivo, we performed eye-wipe studies in male rats. Following acclimation, rats were pretreated with OSCC CM or control UCM followed immediately by 0.01% CAP. A significant increase in nocifensive behaviors was noted in rats pretreated with CM compared to UCM and compared to CAP alone (Figure 1d; *p* < 0.01 and *p* < 0.05, respectively). 

The same set of experiments was performed using MO to evaluate if OSCC-secreted factors sensitize TRPA1 channels. TG cultures pretreated with OSCC-derived CM followed by MO were sensitized to MO, resulting in a significant increase in CGRP release compared to UCM (Figure 1e). Likewise, this effect was reversed by co-treating with RR. To validate TRPA1 sensitization in vivo, eye-wipe studies were performed as described, but using 0.01% MO. Unexpectedly, no sensitization to TRPA1 was evident in vivo. In fact, a trend for a reduced response to MO was seen with both CM (median = 20) and UCM (median = 23) pretreatments compared to MO alone (median = 83), but none were significant; *p* = 0.18 and 0.13, respectively, Mann–Whitney U (Figure 1f).

### 2.2. Advanced OSCC Patients Who Do Not Consume Alcohol Experience More Functional Pain

**Figure 2:**
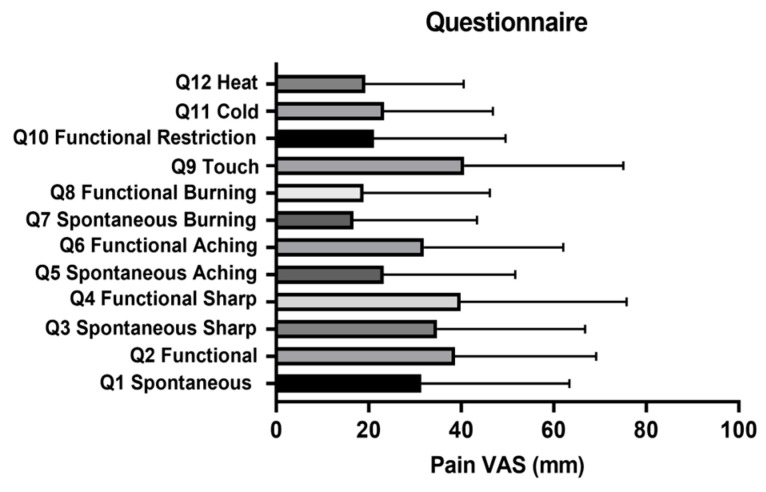
Extended UCSF Oral Cancer Pain Questionnaire. Mean visual analog scale (VAS) pain scores with standard deviation for each of the 12 questions (Q) in the questionnaire.

**Figure 3:**
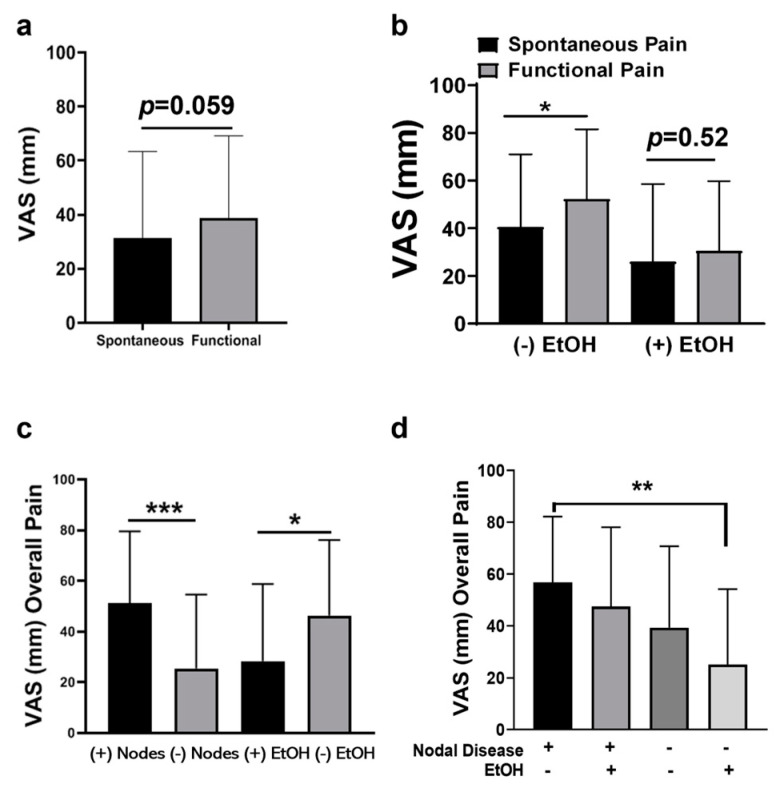
(**a**) VAS scores for spontaneous (Q1) pain vs. functional pain (Q2). (**b**) VAS scores for spontaneous (Q1) pain vs. functional pain (+/−) EtOH consumption. (**c**) VAS scores for overall pain (Q1 and Q2 combined) (+/−) Nodal disease or EtOH consumption. (**d**) VAS scores for overall pain (+/−) EtOH consumption and nodal disease. * *p* < 0.05, ** *p* < 0.01, and *** *p* < 0.001.

Twenty-seven biopsy-proven OSCC patients were enrolled in the study: 15 males and 12 females. Tumor characteristics, demographics, alcohol and tobacco consumption are listed in Table 1. Alcohol consumption was defined as drinking two or more alcoholic beverages a day. The main responses and standard deviations for each question are shown in Figure 2. In contrast to previous reports, we found no differences in reported pain levels between males and females [13,18] and no difference in pain scores based on tumor location [4]. In addition, no differences in pain were identified between Hispanic and Non-Hispanic White patients regardless of disease stage. Similar to previous reports, we saw a trend in increased functional pain scores (Q1) compared to spontaneous pain scores (Q2), $\bar{x}\;$= 38.60 vs. 31.37, respectively, *p* = 0.059, Wilcoxon matched-pairs sign rank test (Figure 3b). However, when evaluated based on alcohol consumption, functional pain ($\bar{x}\;$= 52.30) was significantly greater than spontaneous pain ($\bar{x}\;$= 40.40) in subjects who did not consume alcohol (Figure 3b; *p* < 0.05). In contrast, subjects who consumed alcohol showed no difference in functional pain and spontaneous pain ($\bar{x}\;$= 26.06 vs. 30.59, respectively, *p* = 0.52, Wilcoxon matched-pairs sign rank test). While these levels were lower than those in subjects who did not drink alcohol, they were not significantly lower (Figure 3b).

The overall pain scores (defined as total of spontaneous pain (Q1) and pain on function (Q2) combined) were increased in subjects with advanced stage disease (stages 3 and 4, n = 15) compared to early stage disease (stages 1 and 2, n = 12) based on the TNM classification; however, none were statistically significant. When stratified by the presence of nodal disease, subjects with nodal disease (n = 11) had significantly more overall pain than node negative subjects (n = 16; Figure 3c; *p* < 0.001).

### 2.3. OSCC Pain Is Attenuated by Alcohol Consumption

Of the 27 participants, 17 reported actively using alcohol while 10 reported no alcohol consumption. Rather unexpectedly, when stratified by alcohol consumption, subjects that consumed alcohol reported less overall pain than subjects who did not consume alcohol (Figure 3c; *p* < 0.05). Furthermore, subjects with nodal disease who did not consume alcohol reported the highest levels of overall pain. This was significant compared to node negative subjects who did consume alcohol (Figure 3d; *p* < 0.01). 

**Figure 4:**
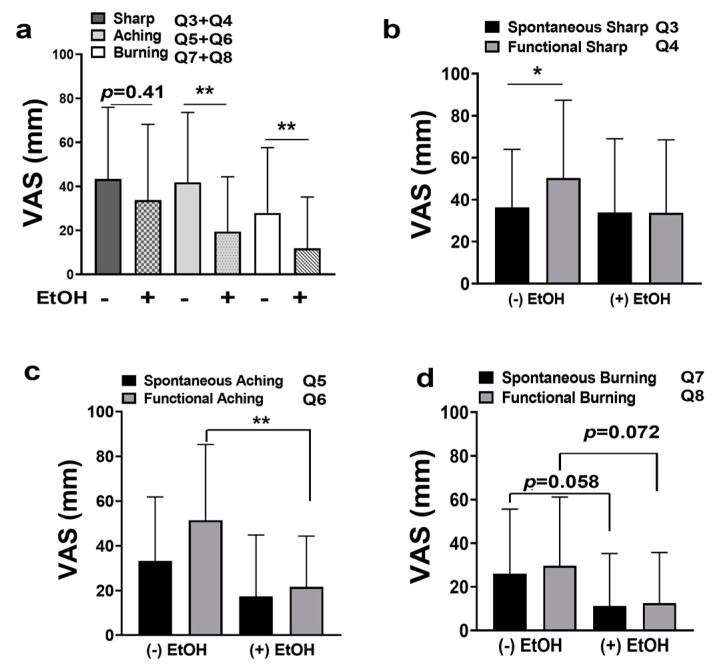
(**a**) VAS scores for total sharp pain (Q3 and Q4 combined), total aching pain (Q5 and Q6 combined), and for total burning pain (Q7 and Q8 combined) (+/−) EtOH consumption. (**b**) VAS scores for spontaneous sharp pain (Q3) vs. functional sharp pain (Q4) (+/−) EtOH consumption. (**c**) VAS scores for spontaneous aching pain (Q5) vs. functional aching pain (Q6) (+/−) EtOH consumption. (**d**) VAS scores for spontaneous burning pain (Q7) vs. functional burning pain (Q8). * *p* < 0.05, and ** *p* < 0.01.

When pain was broken down into categories (sharp, aching, and burning), additional differences were identified. Interestingly, subjects who consumed alcohol reported less pain in all three categories compared to non-drinkers (Figure 4a–d). The most intense types of pain reported were sharp pain, followed by aching pain, and lastly burning pain in both groups (+/−) alcohol consumption. In particular, subjects who consumed alcohol reported significantly less aching pain and burning pain (Figure 4a, *p* < 0.01) and demonstrated reduced levels of sharp pain that were not significant (median = 22.5 vs. 43.0; *p* = 0.41, Mann–Whitney U).

**Figure 5:**
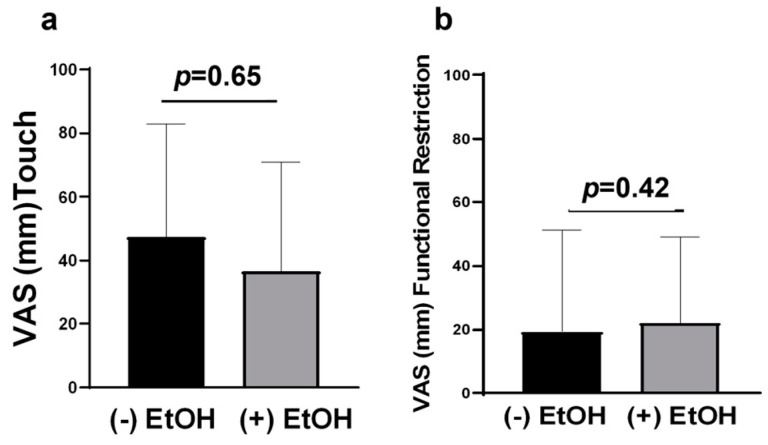
(**a**) VAS scores for pain in response to touch (Q9) (+/−) EtOH consumption; $\bar{x\;}$ = 36.65 (+) EtOH vs. 47.40 (−) EtOH. (**b**) VAS scores for functional restriction (Q10) (+/−) EtOH consumption; $\bar{x\;}$ = 22.12 (+) ETOH vs. 19.40 (−) EtOH.

The analysis of spontaneous and functional sharp, aching, and burning pain levels revealed that participants who consumed alcohol reported no increase in pain levels when in function compared to their reported spontaneous pain levels regardless of pain category (Figure 4b–d). Conversely, participants who did not consume alcohol reported significantly greater levels of functional sharp pain compared to spontaneous sharp pain (Figure 4b, *p* < 0.05). However, both drinkers and non-drinkers reported no significant increase in functional burning pain compared to their reported spontaneous burning pain levels (Figure 4d). When analyzing drinkers’ vs. non-drinkers’ pain levels, subjects that consumed alcohol reported less spontaneous aching pain and significantly less functional aching pain compared to non-drinkers (Figure 4c, *p* < 0.01). In addition, subjects that consumed alcohol demonstrated a trend in reduced spontaneous burning pain compared to non-drinkers (median = 0 vs. 22, respectively; *p* = 0.058, Mann–Whitney U) and a trend in reduced functional burning pain compared to non-drinkers (median = 0 vs. 24, respectively; *p* = 0.072, Mann–Whitney U; Figure 4d). Finally, alcohol consumption was not associated with functional restriction or pain in response to touch (Figure 5a,b).

### 2.4. OSCC Patients Experience Thermal Allodynia

**Figure 6:**
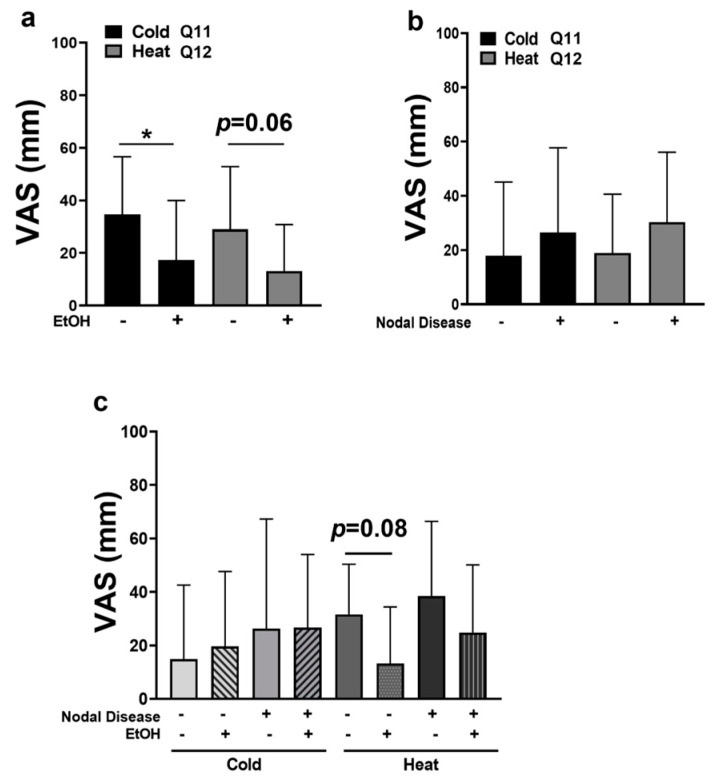
(**a**) VAS scores for cold (Q11) and heat (Q12) allodynia (+/−) EtOH consumption. (**b**) VAS scores for cold (Q11) and heat (Q12) allodynia vs. nodal status. (**c**) VAS scores for cold and heat vs. both EtOH consumption and nodal status; * *p* < 0.05.

To our knowledge, this is the first clinical study evaluating OSCC-patient-reported pain in response to thermal changes. Indeed, pain in response to cold and heat was demonstrated in all subjects. Subjects who consumed alcohol reported significantly less cold related pain (Figure 6a; *p* < 0.05) and tended to have less heat related pain compared to subjects who did not consume alcohol (median = 3 vs. 25.5, respectively; *p* = 0.06, Mann–Whitney U).

Similar to sharp, aching, and burning pain, node-positive subjects reported more pain in response to cold and heat compared to node-negative subjects; however, this was not statistically significant (Figure 6b). The evaluation of thermal allodynia in the context of both nodal disease and alcohol consumption was limited due to the small sample size. However, we noted that subjects with nodal disease displayed cold-related pain that was greater than node-negative subjects, regardless of alcohol consumption. On the other hand, heat allodynia tended to be reduced in node-negative subjects who consumed alcohol compared to node-negative non-drinkers (median = 8 vs. 40, respectively, *p* = 0.08, Mann–Whitney U). Indeed, node-negative subjects who consumed alcohol reported the lowest VAS scores for heat allodynia (Figure 6c). Lastly, node-positive drinkers reported the highest VAS scores for heat allodynia, but this finding was not significant. Future studies utilizing a larger cohort are needed to fully ascertain the interaction between alcohol consumption and nodal disease in thermal sensitivity.

### 2.5. Tobacco

**Figure 7:**
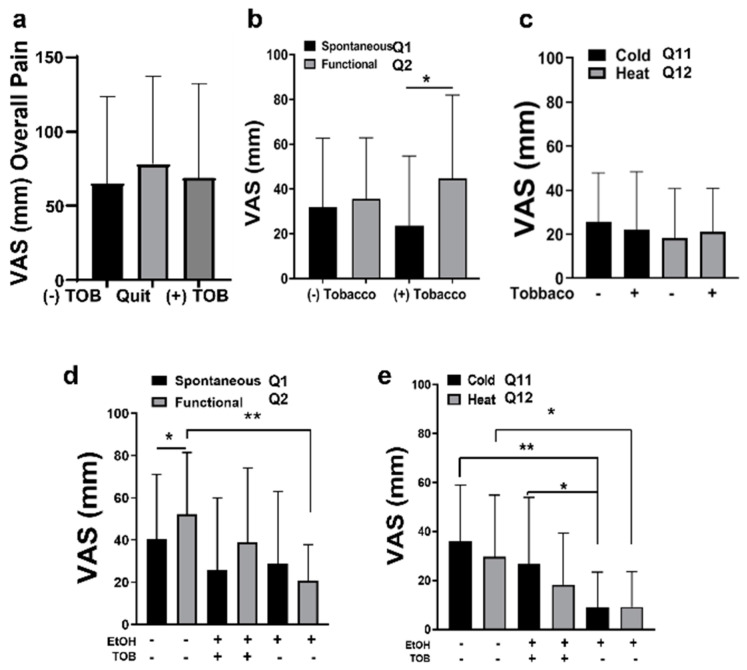
(**a**) VAS score for overall pain (Q1 + Q2) in subjects with a history of tobacco, who had quit tobacco, and who were currently using tobacco. (**b**) VAS scores for spontaneous pain (Q1) and functional pain (Q2) (+/−) smoking tobacco. (**c**) VAS scores for cold and heat allodynia (+/−) smoking tobacco. (**d**) VAS scores for spontaneous pain (Q1) and functional pain (Q2) vs. smoking tobacco and EtOH consumption (+/−). (**e**) VAS scores for cold and heat allodynia vs. smoking tobacco and EtOH consumption. * *p* < 0.05 and ** *p* < 0.01.

Unlike other studies, we found no differences in overall reported pain levels (Q1 + Q2) between subjects who were actively smoking tobacco and subjects who had quit or with no history of smoking (Figure 7a).

When analyzing spontaneous and functional pain, there was no significant difference between smokers and non-smokers (Figure 7b). However, subjects who smoked tobacco had significant increased pain scores when in function compared to their spontaneous pain scores (Figure 7b; *p* < 0.05). This was the only indicator that tobacco use may increase functional pain in oral cancer patients. Lastly, no differences in thermal allodynia were noted between smokers and non-smokers (Figure 7c). 

When analyzing thermal allodynia in relationship to both alcohol consumption and smoking tobacco, a trend of decreased pain scores remained for thermal allodynia in subjects who consumed alcohol. Notably, subjects who consumed alcohol and used tobacco reported significantly higher pain scores in response to cold vs. subjects who consumed alcohol and did not smoke tobacco (Figure 7e; *p* < 0.05). This was our only indication that tobacco use may increase pain in response to thermal changes. These analyses of both alcohol and tobacco usage together were somewhat limited by the small sample sizes. Indeed, this study was powered to evaluate the effects of each variable such as alcohol or tobacco use independently. However, the sample size limited our ability to fully ascertain the effects of both variables together on oral cancer pain. Notably, nine out of ten subjects who reported no alcohol consumption also reported no history of smoking tobacco. Only one subject reported using tobacco but not alcohol, and thus was excluded from these analyses. Therefore, additional studies with larger cohorts are needed to ascertain the effects of both tobacco and alcohol consumption on oral cancer pain.

## 3. Discussion

Aside from death, pain is the primary concern for oral cancer patients. Studies confirm that oral cancer is one of the most painful cancer types, with opioids being the mainstay treatment. However, oral cancer patients quickly develop tolerance [13,19], leaving them with few therapeutic options while battling this deadly disease. Connelly et al. (2004) developed and validated the UCSF Oral Cancer Pain Questionnaire as a tool to quickly assess oral cancer pain levels and assist with pain management decisions [13,14]. This questionnaire is employed routinely to evaluate oral cancer pain. Using an expanded version of the questionnaire that included additional questions related to burning pain, heat, and cold, we assessed oral cancer pain levels in relation to a number of variables that may mediate pain; ethnicity, gender, tumor staging, nodal disease, alcohol consumption, and tobacco usage. Some limitations to the study included a low sample of subjects who used tobacco but not alcohol, and the lack of objective thermal testing. Notably, we initially performed patient thermal quantitative sensory testing; however, we quickly determined that this was not tolerated by oral cancer patients. Therefore, this study only assessed patient-reported pain through the use of the questionnaire.

Our patient population was 64% Hispanic White and 25% non-Hispanic White, 7% African American and 3% Asian. Therefore, the ethnicity of study participants was largely either Hispanic White (n = 15) or non-Hispanic White (n = 12). We found no differences in pain levels between these two patient populations. 

While male patients are affected twice as frequently as females with OSCC, we detected no differences in pain scores between genders (n = 15 males and n = 12 females). Indeed, female patients have been confirmed to experience more extra-oral pain conditions than males; however, no difference was detected in this analysis of oral cancer pain. Other studies indicate gender differences in oral cancer pain, but the findings are inconsistent. Some studies found that males experience more oral cancer pain than females [4,13]. Conversely, Sheff et al. (2018) and Reyes-Gibby et al. (2011) found that females experience more oral cancer pain than males [18,20]. Sato et al. (2010), found no gender differences for oral cancer pain. Hence, it is unclear if gender truly plays a role in oral cancer pain. To date, there are no known mechanisms of OSCC oncogenesis related to sex hormones to explain differences in incidence between genders. Likewise, very few studies investigate mechanisms that regulate gender differences in oral cancer pain. A more recent study by Scheff et al. (2018), found that tumor-bearing male mice demonstrate a neutrophil-mediated endogenous analgesic mechanism that is not found in tumor-bearing female mice; still, similar studies have not been performed in OSCC patients [20]. Taken together, it remains unclear if there are sex differences in oral cancer pain. Larger mechanistic studies in oral cancer patients are necessary to confirm these findings and delineate possible mechanism(s).

Previous reports also indicate that patients with advanced stage disease and/or nodal disease experience more pain than those with early-stage disease [13,18]. We also observed a trend for lower pain scores in early-stage disease when stratified by TNM staging; however, the difference was not significant. When stratified by the presence of nodal disease, a significant increase in pain levels was detected in node-positive subjects. 

This study demonstrates, for the first time, that oral cancer patients report thermal allodynia to both cold and heat. We also demonstrate that both TRPV1 and TRPA1 channels are sensitized to tumor-secreted factors in vitro. Surprisingly, only TRPV1 was found to be sensitized to tumor-secreted factors in rat models of orofacial pain. This may be due to the short time of exposure to CM prior to MO, or lesser concentrations/instability of TRPA1 activators in the CM to detect in vivo effects. In addition, repeated exposures to MO or very high concentrations of MO are known to desensitize TRPA1 channels [21,22,23]. Given that both the CM and UCM induced no nocifensive behaviors and very low concentrations of MO were used in this study, it is unlikely that TRPA1 desensitization explains our findings. Thus, additional studies are necessary to fully understand the effects of OSCC on TRPA1 activity. 

Ruparel et al. (2015) determined that OSCC-secreted lipids induce thermal and mechanical allodynia and nocifensive behaviors in rat behavioral models [24]. Notably, TRPV1 antagonists inhibited thermal allodynia and nocifensive behaviors with no effect on mechanical allodynia. Therefore, it may be that thermal allodynia is mediated by both TRPA1 and TRPV1. In contrast, nocifensive and mechanical allodynia in response to tumor secreted factors may be mediated by additional channels that are yet to be identified. 

Alcohol is a known agonist for TRPV1 channels, yet our findings demonstrate that chronic alcohol consumption is associated with reduced thermal allodynia in oral cancer patients. This is consistent with alcohol-induced peripheral neuropathy, in which patients demonstrate reduced sensitivity to vibration and reduced proprioception to touch, heat, and cold [25]. Alternatively, alcohol may desensitize these channels or alter the central processing of these afferent inputs. Studies evaluating fetal ethanol exposure demonstrate that ethanol attenuates oral aversiveness to CAP, but not MO in adolescent rats [26]. Certainly, further studies are required to more fully evaluate the effects of chronic alcohol consumption on TRP channel activity in OSCC patients. 

Most importantly, we determined that chronic alcohol consumption is associated with a reduction in nearly all types of oral cancer pain, including pain in function, aching and burning pain, pain in response to cold, and pain associated with nodal disease. Given that pain is the primary factor that causes most oral cancer patients to seek treatment, the use of alcohol may actually be a contributing factor to late detection or seeking care at later disease stages. Indeed, 70% of all newly identified oral cancers are already in advanced stages, and chronic alcohol consumption is a key risk factor to developing oral cancer. Notably, this study did not find an association of alcohol consumption with late-stage diagnosis. Nearly half of subjects with late diagnosis (43%) reported not using alcohol. Larger scaled studies are therefore needed to fully delineate the role of chronic alcohol consumption as it relates to pain perception and late diagnosis of OSCC.

## 4. Materials and Methods

### 4.1. Human OSCC Cell Lines

Human primary OSCC cell lines Cal-27, SCC-4, and SCC-25 were obtained from the American Type Culture Collection, (ATCC, Manassas, VA, USA). HSC3 cells were kindly provided by Dr. Brian Schmidt (New York University College of Dentistry, New York, NY, USA). Cell lines were authenticated within six months of experiments by Genetica DNA Laboratories (Cincinnati, OH, USA). Cells were maintained in Dulbecco’s Modified Eagle’s Medium (DMEM, Gibco; Thermos Fisher Scientific, Inc., Waltham, MA, USA) supplemented with 10% fetal bovine serum (FBS) and 1% penicillin/streptomycin and maintained at 37 °C under 5% CO^2^.

### 4.2. Reagents

Cell culture studies used 10 M stock solutions of capsaicin (CAP) and mustard oil (MO; Sigma-Aldrich, St. Louis, MO, USA) diluted 100% EtoH. Ruthenium red (RR; Sigma-Aldrich, St. Louis, MO, USA) was prepared as a 100 mM stock in sterile water. Stock solutions of CAP and MO for rat orofacial pain model were diluted to 0.01% (*w*/*v*) in 100% sterile saline just prior to use. CM was collected from OSCC cell lines plated at 75% confluency, washed with 1× phosphate-buffered saline, and then incubated with freshly prepared serum free DMEM for 24 h as previously described [27]. Once collected, CM was centrifuged (1 g for 5 min) to remove free-floating cells and the remaining media was transferred to a sterilized microfuge tube and stored immediately in a −80 °C freezer, and thawed just prior to use. Freshly prepared serum-free DMEM served as the UCM control just prior to use. 

### 4.3. Rat Trigeminal Ganglia (TG) Primary Culture

Six TGs from three rats were dissected immediately following decapitation and processed as previously described [27]. Briefly, TGs were immediately placed in ice-cold calcium and magnesium-free balanced Hanks solution (Gibco) and washed 3× with Hanks balanced salt solution (HBSS). They were then treated with 5 mg/mL collagenase (Worthlington Biomedial, Lakewood, NJ, USA) for 30 min and 0.1% trypsin for 15 min followed by homogenization. TGs were then centrifuged at 2000 rpm for 2 min and resuspended in DMEM containing 1× penicillin/streptomoycin, 1× L-glutamine, 10% FBS, mitotic inhibitor (0.3 mg/mL FDU and 0.7 mg/mL uridine), and 10 ng/mL nerve growth factor (NGF, Harlan, Indianapolis, IN, USA). Cells were plated on one 24-well poly-D-lysine-coated plate (BD Biosciences, Bedford, MA, USA), yielding approximately 8000 cells per well. Media were replaced after 24 h incubation and again at 48 h incubation. Calcitonin gene-related peptide (CGRP) release experiments were performed on day 5–7 of primary cultures. 

### 4.4. CGRP Release Assay and iCGRP Radioimmunoassay (RIA)

CGRP release was performed as previously described [27]. Primary TG cultures (n = 4 per group) were washed 3× with Hanks solution and then allowed to incubate for 15 min to collect a baseline sample. Initial experiments evaluated the direct effect of CM from OSCC cell lines by treating with CM or UCM for 30 min. To determine if CGRP release was due to cation channel activation, TGs were also pre-treated with 10 µM RR or vehicle control (Hanks solution) for 15 min followed by co-treatment with CM. To determine if CM sensitized TRPV1 in primary TG cultures, cells were co-treated with CM and CAP (10 nM) with and without RR (10 µM). To confirm that neurons were vital following these treatments, cells were washed and incubated in Hanks solution for 15 min and a recovery sample was collected. 

A previously used primary antibody against CGRP (diluted 1:1,000,000, kindly provided by Dr. M.J. Iadarola, NIH) was added to the tubes containing the supernatant from baseline, treated, and recovered primary TG cultures and incubated for 24 h at 4 °C. Then, 100 µL of [I^125^]-Tyr_o_-CGRP_28–37_ (at approximately 25,000 CPM) and 50 µL of goat anti-rabbit anti-sera coupled to ferric beads (Qiagen, Valencia, CA, USA) were added to the tubes and incubated for 24 h at 4 °C. Immunomagnetic separation was used to halt the assay, and the unbound tracer was aspirated from all tubes. CM and UCM with or without CAP and RR were tested for interference with RIA. The same protocol was performed using MO (0.01%) with and without RR to determine if TRPA1 channels were sensitized as well.

### 4.5. Eye-Wipe Testing

All procedures for animal studies were approved by the UT Health San Antonio (UTHSA) Institutional Animal Care and Use Committee (IACUC) and followed the NIH Guidelines for the Care and Use of Laboratory Animals. In addition, all rat studies complied with the Animal Research: Reporting of In Vivo Experiments (ARRIVE) guidelines and the 2013 American Veterinary Medical Association (AVMA) euthanasia guidelines. Six-week-old male Sprague Dawley rats, weighing approximately 300 g (Envigo Laboratories), were provided with a 12 h light/12 h dark schedule at a controlled temperature and humidity, with food and water available ad libitum. Rats were acclimated for two weeks prior to study initiation. Rats were placed in a temperature-controlled (22–25 °C) behavioral laboratory in individual mirrored testing boxes (30 × 30 × 30 cm) in which they were allowed to acclimate for at least 1 h. One drop (40 µL) of a solution of 0.01% (*w*/*v*) CAP or MO, in sterile saline, was dropped onto one eye of each freely moving animal (n = 6 per group), as described previously (14, 15). Pre-treatment with one drop of UCM or CM from OSCC cell lines was also performed, followed immediately with one drop of CAP treatment. The time spent grooming or closing the affected eye was recorded for a total of 5 min, with the observers blinded to the treatment allocation groups.

### 4.6. Expanded UCFS Oral Cancer Pain Questionnaire

We utilized the UCSF Oral Cancer Pain Questionnaire for use in this study. This questionnaire consists of 8 questions related to spontaneous pain and functional pain (e.g., pain when eating and speaking) in addition to different types of pain, including sharp and aching pain. Pain in response to touch (i.e., mechanical pain) and functional restriction are also evaluated. We extended the questionnaire by adding four additional questions regarding pain with cold exposure, pain with heat exposure, and spontaneous and functional burning pain. These questions were rated by the patient on a visual analog scale of 0 to 100 mm. Only patients with biopsy-proven OSCC who had not received any prior treatment or analgesics were included in the study (n = 27). Patients completed the questionnaire at the appointment following their biopsy, which occurred from 10 to 21 days post-biopsy at the UTHSA Otolaryngology clinic. Data collection included the patients’ gender, age, TNM staging (according to *the American Joint Committee on Cancer Staging Manual* [28]), and a history of alcohol and tobacco consumption. No subjects reported initiating an alcohol or tobacco habit after their oral cancer diagnosis. Subjects who reported an alcohol habit prior to their oral cancer diagnosis and continued to drink two alcoholic drinks or more a day were categorized as current alcohol consumers. Subjects who reported a tobacco habit prior to their oral cancer diagnosis and currently smoked at least three cigarettes a day were categorized as current tobacco consumers. Subjects who had quit tobacco at least three months prior to enrollment in the study were considered to have quit. The questionnaire responses were scored and compiled with patient data. This study was approved by the UTHSA Institutional Review Board (IRB), and informed consent was obtained from all participants prior to the study.

### 4.7. Statistical Analysis

GraphPad Prism 8 (San Diego, CA, USA) was used for all statistical analyses. Experiments were performed at least in triplicate, and results are represented as means ± standard error of the mean (SEM) for parametric tests and as medians for nonparametric tests. One-way analysis of variance (ANOVA) was performed for all CGRP release assays and behavioral testing. Student’s t-test was used to analyze differences between two treatments in CGRP release assays. The Mann–Whitney U test was also used to analyze differences between two treatments in the behavior studies. The Mann–Whitney U test and Wilcoxon matched-pairs signed ranks test were performed to analyze the expanded UCSF Oral Cancer Pain Questionnaire data. In all experiments, a *p*-value less than 0.05 was considered statistically significant.

## 5. Conclusions

OSCC patients experience thermal allodynia to both cold and heat, which may be mediated by TRPA1 and TRPV1. Chronic alcohol consumption attenuates multiple types of oral cancer pain. Future studies focused on TRP channel expression and activity in the context of chronic alcohol consumption are needed to ascertain the potential effects for delayed care. In summary, the identification of TRPV1 and TRPA1 channels as biological targets to treat oral cancer pain, and chronic alcohol consumption as a confounding factor in patient-reported pain, which may mediate TRPV1 and TRPA1 activity, is critical to developing non-opioid drugs to treat oral cancer pain.

## Figures and Tables

**Table 1:** Patient Demographics, Tumor Staging, EtOH, and Cigarette Tobacco Usage.

Patient	TNM	Nodal Disease	Location	Sex	Age	Race	EtOH	Tobacco
1	T 2 N1 M0	Yes	Tongue	M	47	Non-Hispanic White	Yes	Yes
2	T 4 N2 M0	Yes	BOT & OP	M	57	Non-Hispanic White	Yes	Quit
3	T2 N1 M0	Yes	FOM	M	62	Non-Hispanic White	Yes	Quit
4	T4 N1 M0	Yes	Buccal Mucosa	M	44	Hispanic White	Yes	Yes
5	T2N1M0	Yes	BOT	M	46	Hispanic White	Yes	Yes
6	T2 N1 M0	Yes	Max. alveolar ridge, Palate, BOT	M	53	Non-Hispanic White	Yes	Yes
7	T2 N2 M0	Yes	Tongue	M	63	Non-Hispanic White	Yes	Quit
8	T3N0M0	No	Soft Palate	M	73	Hispanic White	Yes	Yes
9	T4 N0 M0	No	Mand. alveolar ridge	M	58	Hispanic White	Yes	No
10	T2 N0 M0	No	Tongue	M	52	Hispanic White	Yes	No
11	T4 N0 M0	No	Tongue and FOM	F	81	Hispanic White	Yes	Quit
12	T1 N0 M0	No	Tongue	F	49	Non-Hispanic White	Yes	Yes
13	T1 N0 M0	No	Tongue	M	62	Non-Hispanic White	Yes	Yes
14	SCC In Situ	No	Tongue	M	53	Hispanic White	Yes	Yes
15	T2 N0 M0	No	Buccal Mucosa	M	67	Non-Hispanic White	Yes	Quit
16	T2 N0 M0	No	Tongue	F	79	Hispanic White	Yes	Quit
17	T1 N0 M0	No	FOM	F	75	Non-Hispanic White	Yes	Quit
18	TxN1M0	Yes	Tongue	F	67	Non-Hispanic White	No	No
19	T1 N2 M0	Yes	Palate	M	58	Non-Hispanic White	No	Yes
20	T4 N1 M0	Yes	Buccal mucosa	F	81	Hispanic White	No	No
21	T1 N1 M0	Yes	Tongue	F	50	Non-Hispanic White	No	No
22	T1 N0 M0	No	Tongue	F	42	Hispanic White	No	No
23	T1 N0 M0	No	Tongue	F	77	Hispanic White	No	No
24	Invasive SCC	No	Max alveolar ridge	F	78	Non-Hispanic White	No	No
25	T4 N0 M0	No	Tongue	M		Hispanic White	No	No
26	T4 N0 M0	No	Mandible	F	93	Non-Hispanic White	No	No
27	T2 N0 M0	No	Tongue	F	57	Non-Hispanic White	No	No

## Data Availability

Data is contained within the article.
